# Community Perspective on Policy Options for Resettlement Management: A Case Study of Risk Reduction in Bududa, Eastern Uganda

**DOI:** 10.1371/currents.dis.49e8e547de25ca1c1f9edbbfc8b9efa5

**Published:** 2018-07-26

**Authors:** Stella Neema, Grace Mongo Bua, Doreen Tuhebwe, Julius Ssentongo, Nathan Tumuhamye, Roy William Mayega, James Fishkin, Lynn M Atuyambe, William Bazeyo

**Affiliations:** College of Humanities, Makerere University, Kampala, Uganda; School of Public Health-Resilient Africa Network, College of Health Sciences, Makerere University, Kampala, Uganda; Department of Health Policy, Planning & Management, School of Public Health, College of Health Sciences, Makerere University, Kampala, Uganda; School of Public Health-Resilient Africa Network, College of Health Sciences, Makerere University, Kampala, Uganda; Department of Epidemiology and Biostatistics, School of Public Health-Resilient Africa Network, College of Health Sciences, Makerere University, Kampala, Uganda; Department of Epidemiology and Biostatistics, School of Public Health, College of Health Sciences, Makerere University, Kampala, Uganda; Stanford University Center for Deliberative Democracy, ResilientAfrica Network; Department of Community Health and Behavioural Sciences, School of Public Health, College of Health Sciences, Makerere University, Kampala, Uganda; Department of Disease Control and Environmental Health, School of Public Health, College of Health Sciences, Makerere University, Kampala, Uganda

## Abstract

Introduction: Despite existing policy actions on Disaster Risk Reduction (DRR), many community members in Bududa still continue to settle in high-risk areas re-zoned for nonsettlement. There seems to be an apparent information asymmetry on expectations between the community and Government. The challenge then is ‘*how to consult communities and seek their opinion in an adequately representative unbiased way*’. This paper sets out to explore policy options on resettlement management as a DRR approach and how engaging with communities in a public discourse using the Deliberative Polling (DP) approach; to obtain their opinions and insights on these policy issues, revealed underlying challenges to policy implementation.

Methods: A qualitative study was conducted in Bududa in eastern Uganda with fourteen group discussions; comprising 12-15 randomly assigned participants of mixed socio-economic variables. Trained research assistants and moderators collected data. All discussions were audio taped, transcribed verbatim before analysis. Data were analyzed using latent content analysis by identifying codes from which sub-themes were generated and grouped into main themes on policy options for resettlement management.

Results and Discussion: We used Deliberative Polling, an innovative approach to public policy consultation and found that although the community is in agreement with most government policy options under resettlement management, they lacked an understanding of the rationale underlying these policy options leading to challenges in implementation. The community members seemed uncertain and had mistrust in government’s ability to implement the policies especially on issues of compensation for land lost.

Key Words: Policy, Deliberative Polling, Climate change, risk-reduction, landslides, Uganda

## Introduction

Disaster incidents are on the increase globally in frequency, intensity and duration especially in the advent of climate change/variability manifested as floods, landslides, drought and glacial runoffs among others 1. This has been worsened by the unpredictable nature of these events. Climate variability is attributed directly or indirectly to human activity that alters the composition of the global atmosphere 2.

In Uganda, landslides are one of the devastating effects that have been faced due to climate variability. Landslides usually occur in hilly terrain and are triggered by persistent rainfall 3^,^^4^. Bududa district of Uganda has a history of landslides, attributed to the hilly terrain of Mt Elgon. Bududa receives an average precipitation of above 1, 500 millimeters (mm) per year (meaning it basically rains every day) triggering landslide occurrence 3. This is worsened by the ever-increasing population which puts much pressure on the land 3. The current population is about 227,400 inhabitants with a population density of 906.7 persons per square kilometre, four (04) times higher than the National average, making it the most densely populated region in Uganda 6. The occupants exploit the slopes of Mt Elgon for settlement and agriculture often causing land degradation 7^,^5.

The most devastating landslide in Uganda occurred on 1st March 2010 in Bududa District^8^^,^^9^^,^^10^^,^^11^.The landslide was triggered by heavy rains that lasted over three months. The landslide buried three villages in Bududa, killing over 400 and displacing an estimated 5,000 people. The landslide led to an immediate breakdown of water and sanitation systems predisposing affected people to disease outbreaks such as cholera^8^^,^^11^.

Following the landslide, several policy recommendations and options were issued in the Uganda National Policy for Disaster Preparedness and Management (2010) 12. The options included resettlement of affected people, re-zoning of the high risk areas for no settlement, compensation of victims, voluntary relocation and establishment of early warning systems many of which were enforced by the relevant authorities 12

In Bududa, resettlement was applied as a long-term risk reduction solution. It involved resettlement of people away from high-risk areas 12^,^^13^. Resettlement of affected persons from Bududa was implemented by the Government of Uganda 12. Affected communities were relocated to Kiryandongo district, in western Uganda majorly because of the availability of vast lands 10. This also included the displaced persons who were temporarily taking refuge in Internally Displaced Peoples (IDP) camps at Bulucheke sub-County headquarters in Bududa district 10. However, despite the availability of vast land, It is important to appreciate the significant contextual backgrounds and differences between these two districts; socially, culturally and economically 22. Communities in Bududa are used to settling in the highlands as compared to those in Kiryandongo who are used to settling in low lands. The cultural practices of these two peoples are also different; in Bududa the annual male circumcision''Imbalu'', a rite of passage signifying transition of the young boys to manhood is celebrated and held to such high esteem because it constructs the Bagisu identity, while those in Kiryandongo do not practice these cultural practices, making it difficult for the Bagisu to fit in 22. Economically, the people from Bududa are farmers owing mainly to the highly fertile volcanic soils while Kiryandongo has less fertile soils and are mainly mixed farmers but predominantly herdsmen 22.

Currently, after years of implementing the policy on resettlement of people in Bududa, this policy has not yielded the required outputs. Many Bududa community members still continue to settle in high-risk areas rezoned for non-settlement and many previously relocated to Kiryandongo have returned to the same affected landslide stricken area 14.This poses the question: *why has the resettlement policy failed in this vulnerable community?*

One of the reasons for the failure of this policy could be the ineffective consultation of the affected communities prior to the implementation of the policy. The Uganda National Policy for Disaster Preparedness and Management was developed by the government through a process of conducting consultations at all levels using local leaders at community level, through the district leaders to stakeholders at national level 12. However, it seems that there is information asymmetry between the local community and government expectations regarding resettlement as a risk reduction policy. This has contributed to an apparent complacency about the proposed policy measures in these communities.

In many countries, public consultation during the policy making process does not adequately involve the communities right from the initial stages 15. Governments often use subjective assessments of situations to craft policies for risk reduction. Although the bottom-up approach may seem effective in policy formulation, only a selected few in leadership positions are consulted upon 16

In the bottom-up approach currently being used in Uganda, the community members do not have the opportunity to carefully think through the issues, be educated upon and make an informed decision hence the community members lack the right information on issues affecting them. There is a need to bridge this gap in information asymmetry by devising better ways of public consultation.

The challenge then is *‘how to consult the communities and seek their opinion in an adequately representative unbiased way’*. In order to counteract this challenge, we used a Deliberative PollingÒ (DP) approach. DP offers an innovative tool in which a representative sample of the community can be consulted in depth on key issues. It provides representative and informed opinion data, both quantitative and qualitative, about the views of the public once they have really considered the issues 16

Deliberative PollingÒ(DP) in essence assesses the representative opinions of a population 17. The premise of Deliberative Polling is that when policy options are important for a community, then public consultations about them should be representative of the population and thoughtfully based on the best information available 16^,^^18^.

The first ever successful DP in Africa was conducted in Bududa district in the Mt Elgon region on 7th-8th July 2014. In this paper we examine why there are unsuccessful efforts by the government to effectively communicate the rationale underlying the current policy on resettlement. The reasons were derived from consultation with the community using the DP approach.

Ò Deliberative Polling® is a registered trade mark of James S. Fishkin. The trade mark is for quality control and benefits the Stanford Center for Deliberative Democracy.

## Methodology

**Study Location**

This paper focuses on the DP proceedings from Bududa district. Bududa district is located in Eastern Uganda, bordering Kenya to the east, Manafwa district to the south, Mbale to the West and Sironko the north. The district is mostly mountainous with an average altitude of 5,900 ft above sea level. The area has been prone to landslides that have been catastrophic . The population is mainly Lumasaba speaking 19.

**Study Design**

The entire design of the DP process involved both Quantitative and Qualitative methods. The Qualitative methods assessed *whether* there was a policy change in attitudes regarding the policy options both before and after the plenary sessions while the Quantitative methods (handled in a separate research paper) assessed *to what extent* and their levels of significance. *This paper focuses on the discussion before the plenary session*. The group discussions were conducted on the 7th and 8th July 2014 in Bududa District. The plenary session is a session where all participants convene and pose questions from the group discussions to a group of experts and policy makers. It provides a platform for transparency, accountability, knowledge dissemination and learning.

For this paper, the study design was a case study. According to Thomas (2011), "Case studies are analyses of persons, events, decisions, periods, projects, policies, institutions, or other systems that are studied holistically by one or more methods 20. In this instance, the case is the Bududa participants who came for the DP and we put them into group discussions. This is because it is from these group discussions that participants’ opinions regarding the various policy options were captured.

**Participants and sampling**

Participants for this study were recruited by random selection of households and random selection of those within the households 18. The DP participants were originally selected through a three-stage sampling technique. During the first stage, 7 sub-Counties from Bududa district were randomly selected: three sub-Counties from the high-risk areas, two from moderate risk and one from low-risk areas. The sub-Counties were simple randomly selected. In the second selection stage, three parishes from each sub-County were selected using simple random sampling technique and the sample size for the district was then allocated to the 21 parishes proportionate to their population sizes. In the third and final stage, participants aged 18-75 years were randomly selected from the parishes. A list of the households, and their adult occupants in each of the selected parishes was compiled by community scouts identified in the respective parishes and guided participant selection. The selection of the sub-Counties was guided by Bududa District Disaster Management Plan 2013, which stipulates that ten sub-Counties are high risk, five are medium risk and one Sub -County; low risk of landslides. One sub-County of low risk was automatically selected and the remaining fifteen sub-Counties were subjected to a ratio of 1:2 hence two sub-Counties for moderate risk and five sub counties for high risk sub-Counties respectively.

The total sample size for this study was 208 participants. In conducting the Deliberative Poll, the random sample first completed a baseline survey in order to collect information about community perception and ranking of importance on the specific policy proposals from stakeholders. The survey respondents were then invited to participate in a DP meeting to deliberate face to face on their understanding and concerns regarding proposed policy options. Originally, the participants of the group discussions were sampled using simple random sampling at household level in the different communities for a quantitative survey. It is from the DP meeting that we purposively selected participants for the group discussions. A total of 14 group discussions of 12-15 participants each were conducted. Figure 1 shows the schematic illustration of the DP process in Bududa.

**Schematic illustration of the DP process in Bududa.**
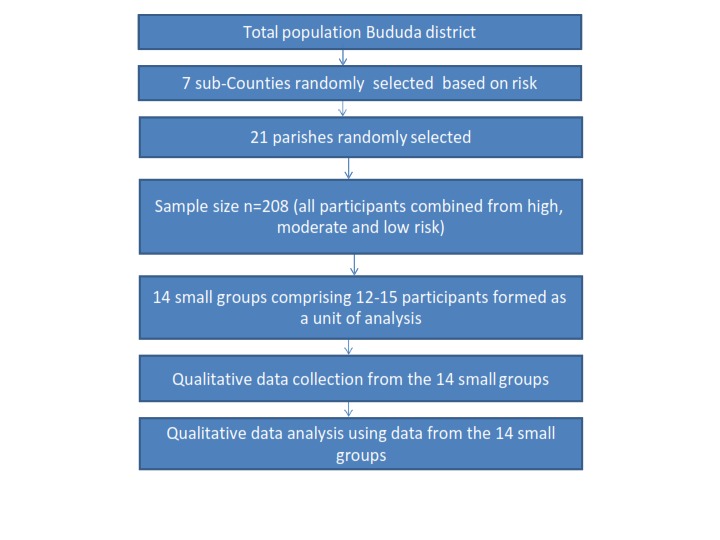

**Data collection methods and procedures**

During the deliberations, participants focused on the pros and cons of the policy proposals and arrived at key questions they wished to pose in the plenary session of experts.

The guide used to moderate the discussion focused on the pros and cons of the policy proposals and arrived at key questions they wished to pose in the plenary session of experts. The guide used to moderate the discussion focused on the policy options around: resettlement management, as an option that can be taken to reduce the damage of landslides. Under resettlement management, the proposals of discussion included re-zoning high risk areas for no settlement, compensation for relocation, resettlement in newly built peri-urban centers, temporary resettlement after a disaster, building an early warning system, supporting local disaster management committees, use of sirens in the early warning system and use of text messages in the early warning system.

**Selection and training of Moderators**

Fifteen research assistants were recruited and trained to facilitate group discussions. The selected research assistants had a minimum of a bachelor’s degree and prior experience in research specifically qualitative research-interview skills. They were knowledgeable in Lumasaba the local language commonly spoken in the district. They were equipped with digital audio recorders to record the group discussions. The training of moderators was jointly conducted by experts from the ResilientAfrica Network, Stanford University and a faculty member from Makerere University School of Public Health.

**Data analysis**

Data collected through the group discussions were transcribed verbatim and those in the local languages translated without altering the meaning. A content analysis approach was used as described by Graneheim and Lundman (2004) 21. Analysis was done in two stages, first, the manifest content analysis (what the text says, deals with the content aspect and describes the visible, obvious components) and then the latent content analysis (what the text talks about, deals with relationship aspects and involves an interpretation of the underlying meaning of the text). The transcripts were read and re-read by the authors who then assigned codes and came up with a coding structure (Open coding). Data meaning units were then aligned under their respective codes. This was followed by axial and selective coding to develop higher codes and categories. Categories were reviewed further to develop overarching themes.

**Ethical considerations**

Ethical approval was obtained from the Makerere University School of Public Health Higher Degrees, Research and Ethics Committee and approval from the Uganda National Council of Science and Technology (UNCST) [study number SS 3532]. Permission to carry out the research was further sought from Bududa District Administration. Verbal consents were obtained from the participants and a request was made to audio-record the discussions. Study objectives, benefits and risks were explained to our respondents. In addition, respondents had the opportunity to ask questions or clarification before consent for the discussion to proceed. All information obtained during the study was treated as confidential.

## Results

In this section we describe the thematic structure of our analysis, showing the main themes and sub-themes regarding policy options for resettlement management. Our key themes were relocation from high to low risk areas, relocation to relatives, compensation for resettlement and risk communication.

**Participants**

We conducted fourteen group discussions where participants were assigned randomly to groups comprising 12-15 participants of mixed gender (58.7% male and 41.3% female); 90% were married,57.7% primary education,10.4% had no education;86.6% were farmers, and the average number of children per woman was 6.3 as shown in Table 1.

**Table 1: Demographic characteristics of study participants:** 

Variable	Number	Percentage (N=208)
Sex:		
Male	122	58.7%
Female	86	41.3%
		
Marital Status:		
Married	187	90.0%
Single	10	5.0%
Separate/Divorced	3	1.5%
Widowed	7	3.5%
		
Highest Level of Education:		
None	22	10.5%
Primary	120	57.7%
O Level	58	27.9%
A Level	2	1.0%
Tertiary	6	2.9%
		
Occupation:		
Farmer	180	86.6%
Professional/technical/managerial	6	3.0%
Entrepreneur(business owner)	7	3.5%
Merchant	2	1.0%
Teacher	4	2.0%
Student	4	2.0%
Other	4	2.0%
		
Average Number of Children:		6.39

The analysis identified within the three policy options for resettlement management as: (i) resettlement with support for livelihood and in the same community, (ii) Modalities of compensation; (iii) risk communication as early warning favourable to save life. These themes are described in detail in the next section of the article. Figure 2 shows the thematic structure of the research findings based on Gioia (2013) 23.

**Thematic structure of the research findings (Gioia ,Corley et al 2013):**
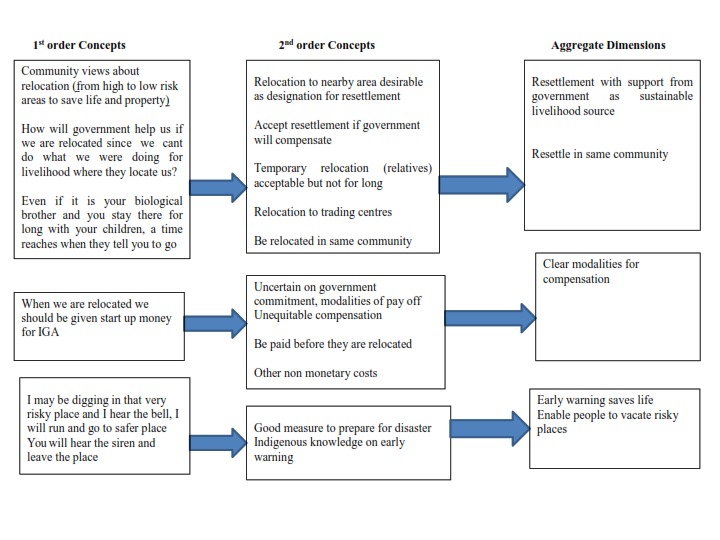

**Relocation from high to low risk areas**

The first main theme was resettlement from high to low-risk areas. Low-risk areas would involve being relocated from the high risk mountainous areas characterized by cliffs, steep colluvial deposits and scars due to previous landslides to low risk areas which are low lying, receive less rain to trigger landslides and relatively safer. These low risk areas mainly have trading centres and are inhabited mainly by relatives. In all the group discussions held, the issue of relocation created mixed feelings. They discussed whether relocation would be temporary or permanent. Participants seemed to weigh the risks of staying in the risky areas and the benefits of relocation.

In general relocation of people who live in hilly risky areas to lower less risky areas was acceptable. This would help them go back to check on their gardens and do some farm work. This was mostly on condition that they would access their gardens or be relocated in areas where they can do farming,''

*I also say that people like us from Bukalasi, say that, when it rains a lot of our lives are always in danger and always leave us very worried. Therefore, I suggest that you people should fight for us a lot. That government should help us to take us in places like this one so that we can avoid being worried all the time when it rains,'' (group 3).*

Saving life and property outweighed clinging to their land. They reported of the many landslides in that area where many people including their relatives lost their lives as illustrated by the quote below:

“*As I talk, in 2010 there was a heavy down pour in the morning and the mudslide covered up my brother’s house and killed five children on spot. It’s good for people to be resettled to other parts of the district, “(Group 6)*.

Participants were affirmative and wanted relocation due to the situation they were in especially those who were in very high risk places such as Buwilimbi parish, Bukibokolo sub-County where they would lose their lives in case of landslides. They noted that preventive relocation would save the government in spending more money to manage disasters. Hence landslide risk significantly affects people’s willingness to resettle. People living in the riskiest areas such as Bukibokolo sub-County in Bududa had a strong willingness to resettle.

That said, most participants were in favour of relocation so long as the government was willing to support them. An assurance of compensation would facilitate their acceptance of relocation. Thus relocation was tagged to compensation of some kind.

“*I think it helps, because if problems befall you, at least you have a starting point, and if the government gives you this assistance, relocation becomes easier. So I'm in support of the idea of relocation,*” *(Group 5)*.

The participants often highlighted that the reasons as to why people resist relocations is because in most cases, they are promised assistance but in the end they get nothing. They were not sure what the funds promised for relocations were used for. Referring to the past experience of those who were relocated in 2011 to Kiryandongo District, people suffered more and some of their land was sold.

“*Now like for us people from Nametsi, we are in area where there were landslides but they carried people and took them to Kiryandongo but for us we refused to go. Now they are yearning to come back and yet some of them have sold off all their land. Where will they start from? Because the conditions are not favourable where they were taken” (group 4)*

The destination for resettlement was discussed extensively and this influences the willingness to be relocated. Most groups preferred to be resettled anywhere in greater Masabaland where they share the same language and culture. They were not in favour of being resettled elsewhere. Social cohesion would be fostered better and they would be able to perform their cultural rituals especially the male circumcision ''Imbalu'' which is a rite of passage to adulthood for this particular ethnic group. Hence, such opinions indicate that an individual’s sense of identity and belonging profoundly discourages people from relocating.

“*For me, I was saying that relocating in Masaba land, within Mbale is better than relocating out (of Masaba land). Because we shall understand lugisu (the local language), we speak, work together and perform our cultural rituals together. And if a problem has befallen us like I may be in Bubulo, I can just walk and come even if I do not have money I can come home,” (Group 4)*

However,a few people, were specific and mentioned that the relocation should be in Bududa low lying areas such as Busanza, Manjiya and Namatyale. They alluded to the fact that Bududa has very fertile soils compared to other districts in greater Mbale.

“*On the issue of being relocated is good but we the people of Bududa, we want to be relocated within, they should not take us to another sub-county or other district because there is a lot of land Bududa like in the low lands of Nalwanza”(Group 1)*

Group participants had many questions related to the type of support provided by the government and how long it would last. Concerns about the ownership of their land in the high risky areas when they leave were raised. They were not sure whether they would still remain with their land after relocation.

“My question is like this, Government thinks of relocating people who are in risky areas, to remove them and take them to other places; if they take them away, who will have authority over the places they have left behind,” (Group 4)

**Relocations to trading centres:** Most participants felt that relocation from risky areas to trading centers was a good idea because trading centers are spacious, safe and accessible to social amenities than rural areas (e.g. clinics, shops). They noted that the services from government and NGOs would reach relocated community better and faster, and all would benefit.

“*I support it because, it will bring development in the trading centres and besides, when government sends assistance, it will be easily accessed by many since they will be in one place,”(Group 5)*

A few of the participants disagreed to be relocated to trading centres because they were of the view that trading centres would be crowded and easily breed diseases such as Cholera that were typical of where they camped during the landslide disasters. Negative influences and behaviours would be acquired from the different people put together especially if there is scarcity of food and other needs.

“*Government should be able to meet our demands but not just making us crowd in one area .Now like the way we are here, the first thing is theft and secondly diseases.So it is better for government provide us with a place where we can temporarily be in case a disaster is about to happen so that we do not crowd in centres and get diseases, “(Group 1)*

Related to the above was the fear of the concentration of so many people from different areas with different behaviours and habits such as those who abuse substances like alcohol and drugs. Such would breed quarrels, conflicts and insecurity. These examples given were a reflection of what happened in the previous landslides when they were relocated to some trading centres.

“*There is something burning in my heart, we were here in 2010-2011 but we suffered due to the crowding of people in one area. There were many diseases and the people whom we left behind who were in good places started stealing our things that we left at our homes. If we are to be relocated it has to be within Bukalasi because there are places that are safe it will be easy for us to go and check on our gardens,” (Group 8)*

Most of the participants, being subsistence farmers, felt that in trading centres they would not have land to graze their cows and goats and to do farming. However those who supported being relocated to trading centres were putting their lives by relocating to a relatively safe area and only using the risky area to farm. Trading centres were preferred because they would be near their ancestral homes than relocation outside the greater Mbale region.

“*I think that if the trading centres are near our original homes, we shall be going to farm and come back to the centres,so that even if the landslides occur, only the food can be affected but not life,” (Group 5).*

**Relocation to relatives**

Although extended families are common, most participants did not favour the idea of relocating to relatives. They felt that they would temporarily stay with their relatives but not for long. They noted that the social support systems have weakened and the hospitality would be abused given the high number household members they have including their domestic animals and other property. They felt they would be a burden to their relatives.

*“I also concur with the last member’s idea even if it is a brother’s home you shared the same breast or even share the same mother, if you go there with your children it reaches a time and he chases you away,” (Group 1).*

Relocating to relatives or friends with their families is something that they were not comfortable with. Most felt they could only stay for a short time until the situation stabilizes. Anticipated family conflicts were some of the inhibiting factors in relocation to relatives and friends. Others mentioned that some of their relatives are poorer than they are and so may not be of much help. A few mentioned that since it would be for a short time to relocate to relatives and friends, they can endure that instead of losing life and property due to landslides.

“*It is good to shift to the relative’s even if you are to quarrel than losing all your entire life and family , it’s good to go and endure and after the disaster you can easily come back home, “(Group 6 )*

A few reported they would be itinerant migrants whereby during the rainy season they migrate to a safe place and go back to their homes during the dry season.

“*So for me, I was suggesting in times of dry seasons, those people should remain there and cultivate their crops but in rainy seasons they should get small rooms for their shelter elsewhere, when rainy season stopped, they go back, that is my opinion, I don’t know whether it helps,” (Group 3).*

**Compensation for resettlement**

Another theme on policy options for resettlement was compensation. In several group discussions participants seemed relatively uncertain regarding compensation by government after relocation from high to low risk areas. The land was valued highly valued and thus compensation was seen as not a feasible option. This led to negative attitude towards compensation for resettlement. They were unclear about the modalities of pay off and this resulted into a prolonged discussions. In fact, they had more questions than answers “ *As we were asking, when you compensate people and leave the hilly areas, does that place remain for the government or for the local people?* They were not sure about ownership of the land the moment they are paid off. They preferred to be compensated with the same amount of land they had before the resettlement.

“*Here we need to agree, but let me ask; when I am being paid, am I paid to leave that place permanently[my land] and it remains free? And the money I am paid, am I allowed to buy a place of my choice or what is it”?* *(Group 2)*

That said, they were positive that it was a good idea if the government paid for their land they left behind in the risky areas. But they were cautious and preferred to get a place to be relocated before they are compensated.

One particular issue that emerged from most groups was the inequitable compensation by government to affected persons which in a way influenced relocation negatively.

''*I may have my coffee and I am earning much from it and when I equate it and see that what they are giving in a year is not equal to what government is giving me, I can refuse to relocate. We also have bananas, yams and many others. These help us a lot in our homes,'' (Group 2)*

Thus, government commitment in compensation was considered crucial for people to accept relocation. Moreover, they articulated the perceived benefits of relocation such as being alive and safe, with less difficulties than staying in the risky areas prone to landslides. However, they noted other negative consequences on people’s lives such loosing cultural and social ties which may not be cost during compensation.

**Risk communication**

An early warning system is a response to an assessment of the risk and it involves monitoring, forecasting, warning, dissemination and communication of warning using a range of media and communication channels. Communities and other key actors should know how to respond promptly to avoid loss of life and adverse effects on livelihoods. Group participants were asked about the systems that can be instituted to warn the residents early enough before landslides strike.

In several group discussions, instituting early warning system was desired by participants. They felt it was a good measure to put in place so that people are aware when a problem is about to happen instead of being caught up by the disaster leading to loss of lives and property

“*For me I support it because it will have helped us so much because you may be in the house and maybe not aware that at this moment a landslide is taking place. But if it (early warning system) sounds like an alarm, or when it (early warning system) sounds like an ambulance, you just know that we have got a problem and we start moving away from that area and relocate to another area,” (Group 9)*

The participants however, reported that traditionally warning systems were in place such as the traditional drum beats that were used to alert people in case of danger, community work or even for festive events as illustrated by the quote below.

“*These things were in place like long ago if somebody died they could easily drum,there was a particular drumming which showed that it is circumcision and there was also a particular drumming which could also alert people to come for drinking. When it drummed, someone could easily tell that there is local brew (alcohol) at this person’s place. So when those things are put in place, one knows if there is a particular kind of drumming, it signifies landslide. People shall always be aware,” (Group 3).*

Drawing on such experiences of community warning systems helps inform and lay the groundwork for the future early warning systems because the early warning systems are able to use both indigenous knowledge and modern knowledge. The community noted that those with their indigenous knowledge know when the risky months are; usually May, September and October when there is a lot of rains.

“*So we know all these periods in our heads but we still suddenly find when it has slid, so it doesn’t help us much especially if you are near such risky areas”. *The participants who were from the high-risk areas prone to landslides gave their real life experience with landslides and so supported the establishment of early warning systems.

“*I support it because for us who live on the hills, now like for me I sleep in between escarpments, there is a hill on this side and one on the other side. There is a time when a landslide occurred across there, we stood on that hill to look at the people at the other end and we were listening as people were shouting, it was dark, what we saw were only torches and we also continued to make alarms so much and the people from the other end continued running and yet this alarming of ours does not help so much. That is why I was saying that if that early warning system is put in place, it will have helped us,” (Group 14)*

The bells and sirens were desired by most groups and that they could be instituted in areas that are risky. The bells and sirens were thought to be good because they were audible enough. However some participants were not sure how these early warning systems could work given that disasters such as floods or landslides happen suddenly. Moreover, they needed to understand the type of sound of the bell that would signify danger.

“*It’s true these early warning systems are good , but we don’t know the devil's plans or those of God. Because when these disasters are going to happen, they don’t inform people, now how will they inform people that a disaster in form of a landslide is going to happen?”(Group 7)*

A few were skeptical about the bells and sirens as the landslide might occur at night when people are asleep and so they may not hear the alarm. Some of the houses are iron roofed and when there is a storm the sound of the siren may not be heard. Or if it is heard people may be in disarray and end up running to where the danger is. They alluded to the fact that the way landslides occur is a process; that it does not happen during the heavy downpour rather it is when the rain is slowing down that the mud slides begin to move down as a mass. So, they felt that the bells and sirens may warn when the landslide is forming and this may be too late as some people would be swallowed on the way to safety. Hence the risk communication system may not be dependable or may not be effectively communicated especially to the most vulnerable populations. In one of the landslide affected area of Nametsi they reported the landslide happened at night.

“*When a landslide is happening, it doesn’t do so during a heavy downpour. It times when the rain is slowing down then the mud slides down. The early warning systems may warn when it happening and when you run out it can find you on the way. For me I support the issue of relocation to safer places than depending on the warning systems,”(group 5)*

“*I have a doubt with regard to that issue because these landslides may occur in the night and usually it happens during heavy stormy rains when the clouds are very heavy and dark in iron sheet roofed houses which are very nosy. For example, in Nametsi the landslide occurred in the night when people were sleeping and even those who moved in the low land, the landslides buried them as well. So our thoughts are really troubled,” (Group 11)*

The participants wanted to know more about the early warning system that would be established in order to act responsibly. They categorically indicated that alarms with no guidance on where to run to will not save the situation in such emergencies.

One of the options mentioned by a few group discussions was the use of short phone messages popularly known as ‘SMS’. This option was found only feasible to a few community members that owned mobile telephone sets. While this communication system had potential to reach out to many people at the same time, a number of issues were raised that rendered it ineffective. Participants raised the problem of low literacy (ability to read and write) as most know only how to receive and make a call by pressing some familiar iconic buttons; phone ownership density where only few people especially men owned mobile phones; low network coverage as well as low battery. At night, most of the phones are switched off for charging rendering SMS unreliable for disaster response.

‘’...*the idea of sending messages on phone is good for like me who have a phone. Once the message is sent and received; that message on phone, you can go to your brother who is in the neighbor hood and inform them about what is bound to happen. It is a very good idea,’’ (Group 1)*

‘’..*the massages sent by phone are good but the problem is not all people have phones and another problem is that some of us may have phones but do not know how to read so even if a message is sent I will not be able to read it.....They just told us that you press here like this, (illustrates), (laughter), and you put on the ear,’’ (Group 1).*

‘’*the way me I see, not all of us have cell phones, and me I really see the best option of reminding us of any problem is the siren but not text messages since we have no phones’’(Group 3)*.

## Discussions

The results show that while the community is in agreement with most of the policy options proposed by the government such as relocation from high to low risk areas including trading centres and to relatives and risk communication including early warning systems, others were in disagreement with the above policy options for the reasons highlighted in the results section. Still,others were uncertain about the proposed policy options e.g compensation for resettlement. The community members also still have mistrust in the ability of the government to fulfill the promises. Moreover, the community members do not understand the *rationale* underlying some of the policy options proposed by government let alone an *understanding* of the proposed policy itself.

Regarding the policy on resettlement, most participants were in favour of relocation as long as the government was willing to support the affected persons through the process. This is in line with Bankoff who noted that communities that are affected by hazards tend to respond by way of helping one another, by providing shelter, food and other necessities with those who have lost their livelihood 24 .

Although in principal, mobility is often understood as a potentially beneficial strategy for vulnerable households, to cope with and reduce exposure to hazards, the exacerbated climatic shocks that have resulted from climate change have rendered resettlement as a core risk reduction strategy as is in the case of Bududa 3. In Uganda, resettlement was done as part of the risk reduction strategy for Bududa District 12.

For its success, the benefits of resettlement have to be clarified to the affected community. Much as some institutions define resettlement as a physical movement , in this community of Bududa, resettlement has not been defined well,hence the question from the community on whether resettlement was permanent or temporary. This speaks to the need for better communication about risk reduction programs. Other questions still unanswered were whether the community members would still own the land in the area where they have been resettled from.

Benefits such as the intention to lessen site-specific vulnerabilities for example in areas like Bududa that are prone to recurrent landslides must be re-laid to the community as was the case in 2008, when a landslide severely affected 85 households in a densely populated and low-income community of Cochabamba city,Bolivia 37. The proposed solution was the relocation of the affected communities from the high risk zone areas 25. Resettlement has also been implemented in areas of civil stiff to reduce risk faced by persons during wars 26

Resettlement as a coping strategy can contribute to income diversification enhancing capacity of households and communities to cope with the adverse effects of environmental and climate change stresses. It also can be a long-term adaptation strategy. The intention is that resettled people will be better off over time as a result of resettlement – according to their own assessment and external expert review 27

In the case study of Vietnam, the outcomes of relocation and resettlement were mixed and it was demonstrated that resettlement programs have the potential to increase resilience and security of vulnerable households 28. However, the question sometimes remains, what do the affected communities perceive as benefits for resettlement? In the case of Bududa district in Uganda, it is unlikely the community perceives the benefits of resettlement such as improved access to public services; protection of the community from environmental shocks and stresses and improved living conditions. This was negated by previous resettlement experiences of 2011 to Kiryandongo. All the groups reported more perceived risks than benefits.

While the resettlement processes have many benefits, resettlement has also been shown to have challenges such as the increased distances thus people need more time to travel to their agricultural fields 28. This has an implication on the consultations that must take place between the state agencies and communities to identify and address the several factors that contribute to the failure of resettlement efforts.

In Bududa community the issue of the type and place of relocation/resettlement came out strongly with some communities preferring to be relocated in the trading centre. This speaks to the adequacy of the relocation site as has been documented elsewhere 29. Officials have to consider the adequacy of relocation site during their planning since the choice of the relocation site could either enable or hinder the resettlement efforts.

In Bududa, the community members preferred to be relocated anywhere in Masabaland in greater Mbale District where they share the same language and culture. Social cohesion would be fostered better and they would be able to perform their cultural rituals especially male circumcision which is a rite of passage to adulthood. This highlights the complexities and enormous challenge in finding suitable sites for relocating disaster-affected communities.That said, with unchecked population growth in the Mt Elgon region intra resettlement may carry a short term relief which is unsustainable in the long run if the livelihood source remains signifcantly agro and eco-system based.

Policy makers need to be aware that unsuitable new sites can lead to lost livelihoods, lost sense of community and social capital, cultural alienation, poverty, and people abandoning the new sites and returning to the location of their original community 8^,^^11^^,^^37^. The economic, social, and environmental costs of relocation should be carefully assessed before the decision to relocate and where is finalized.

In the case of Bududa district in Uganda, following the March 2010 catastrophic landslide, residents were temporarily resettled in IDP camps that led to challenges of poor sanitation, overcrowding and environmental degradation 8. Wisner and colleagues note that choosing inappropriate land for resettlement i.e. if it’s not close to sources of employment, distancing the new site from vital resources etc can lead to the failure of the resettlement efforts 13.

According to Putro (2012), following a large scale mudflow that happened in Sidaorja, Indonesia in 2006, the villagers’ decision-making process on where to resettle was guided by job patterns: 1) workers tended to choose locations near the city center; 2) farmers preferred to move as a group, maintaining their social network with other community members; and 3) traders, self-employed workers, and others lost their jobs and were forced to live in severe hardship because of the relocation 27. This is in line with our study findings in Bududa where the community members preferred to be resettled in areas that were close to their farmland so that they can continue with livelihood activities. Representative community consultations would have brought out these concerns and possibly aided the success of the resettlement policy in Bududa 30

An assurance of compensation is one of the ways that would facilitate community acceptance of relocation. In Bududa, the state agencies have never come out to clearly communicate the compensation terms for relocation of the affected victims. Compensation has been noted to be a major factor in relocation plans by the International Finance Corporation (IFC) 31 Lack of adequate information on compensation and terms of resettlement compromised the trust in the government policies. In times of compensation during resettlement, it is the right of the community to have fair and transparent compensation process.

IFC recommends that the compensation provided should be equal to or above what is required by law and in agreement with host communities on the methodology for calculating compensation. Although compensating for the loss of social capital can be challenging, IFC considers it a key aspect of compensation. Where possible, compensation is provided in forms other than cash so that long-term goals and livelihood improvements can be achieved 31. Given that in Bududa, resettlement meant that the community members had to incur some losses, compensation has to be taken seriously and consulted on.

Another issue that policy makers had to be aware of during this policy implementation in Bududa was the fact that resettlement could lead to some social disruptions such as men losing their social status and or political positions in cases where populations had to be dispersed 32. Such fears that were not identified prior might have contributed to the failure of the policy.

Added to this, most victims, having lost most of their assets in the landslide are literally left with nothing and therefore cannot be in a position to support them to relocate. They will need to be provided with some relief items- beddings, soap, cooking oil, sugar 33. This implies that relocation may be an expensive venture to a victim and this is worth considering by technical persons and policy makers who enforce these policies.

It has also been noted that resettlement is more likely to be successful when communities fully participate in well-planned adequately financed programmes that include elements such as land-for-land compensation, livelihood generation, food security. In other words there is increased chance of success when resettlement is conceived as a sustainable development programme that includes Disaster Risk Reduction (DRR) 13

As we note that resettlement must be part of a holistic risk reduction plan, in highly vulnerable communities, there is need for effective early warning systems that can generate and disseminate timely and meaningful warning information to enable individuals, communities and organizations threatened by a hazard to prepare and to act appropriately and in sufficient time to reduce the possibility of harm or loss 34.

Interestingly, majority of the participants did not trust use of text messaging for early warning because they did not think the mobile phone system was sufficiently reliable. In fact, majority of the members in the group discussions resisted it because of the various reasons as mentioned in the results section. However, use of Short Messaging Service (SMS) for early warning can be a potentially feasible option when the challenges associated with its use are overcome.

It was noted that sirens as an early warning system technology was desired by the people and it means that this acceptability can be leveraged to initiate and scale up the Early Warning Systems (EWS). It is important for the implementing agencies to adhere to standards of cultural sensitivity, acceptability and suitability of the EWS in order to assure sustainability in building EWS. Choosing a warning communication technology is dependent on considering who the recipients are, their location, their activity, the systems they rely on to receive local news and information, any special needs they may have and how they understand and respond to warnings 35

Rogers and Tsirkunov (2010) noted that one critical step is the willingness to act on a warning and take appropriate individual and collective measures to protect lives and poverty 36. So it’s important to have effective warning systems that can engage its expected beneficiaries by raising awareness and knowledge of risks and ensuring that the actions taken are realistic 36. In line with this our study demonstrated that collective traditional warning systems were noted such as drum beats that were used to alert people in case of danger, community work or for festive events.

**Methodological discussion**

The credibility of this study lies in the fact that we used group discussions and a stratified random sampling strategy. It is rare for qualitative work to be conducted with random samples and almost unprecedented for qualitative work to be done where the number of group discussions together is enough to add up to a credible representative sample of the population. We ought to note, however, that while participants from the group discussions were obtained from the 3 zones (low, medium and high risk zones to landslides), participants were heterogeneously composed. Therefore, it was not possible to conduct analysis by the 3 zones. Non-the-less this form composition generated rich and diverse discussion about policy options for resettlement management.

## Conclusions

From the consultations using the deliberative poll method with the community, it can be generally agreed that resettlement is a highly complex issue. Policy makers have to be aware that resettlement and economic displacement of people can have significant adverse impacts on their future life, social fabric and livelihoods. If consultations are not adequately conducted, success determining issues are swept under the carpet. Ineffective consultations can leave the affected community feeling aggravated,hence do not adhere to the “agreed” position because some facts are only known to the technical people at the district level and policy makers in the District.

We recommend that for disaster risk reduction policies, in order to increase community acceptability and successful implementation of the proposed policies there is a need to increase community engagement during the policy formulation process. Deliberative polling presents a new and inclusive community consultation process of obtaining community perspectives for successful policy implementation.

## Data Availabilty

The supplementary raw and analyzed data that support the findings of this study are available in figshare with the identifier data DOI: 10.6084/m9.figshare.5501326 https://doi.org/10.6084/m9.figshare.5501326

## Conflict of Interest

The authors declare that no conflicts of interest exist.

## Funding

This work was supported by the United States Agency for International Development under Makerere University School of Public Health's Resilient Africa Network (RAN) project. The contents of this work are solely the responsibility of the authors and do not necessarily represent the official views of USAID. The funders had no role in study design, data collection and analysis, decision to publish, or preparation of the manuscript.

## Corresponding Authors

Grace Mongo Bua (gracebuamongo@gmail.com; gbua@ranlab.org; gracefridaypreston@gmail.com)
